# ribofootPrinter: A precision python toolbox for analysis of ribosome profiling data

**DOI:** 10.1101/2021.07.04.451082

**Published:** 2025-09-15

**Authors:** Kyra Kerkhofs, Nicholas R. Guydosh

**Affiliations:** 1 Laboratory of Biochemistry and Genetics, National Institute of Diabetes and Digestive and Kidney Diseases, National Institutes of Health, Bethesda, MD, 20892

**Keywords:** Ribosome profiling, reading frame, metagene, pausing, uORF, iORF, dORF, python, MANE, multimapping

## Abstract

Ribosome profiling is a valuable methodology for measuring changes in a cell’s translational program. The approach can report how efficiently mRNA coding sequences are translated and pinpoint positions along mRNAs where ribosomes slow down or arrest. It can also reveal when translation takes place outside coding regions, often with important regulatory consequences. While many useful software tools have emerged to facilitate analysis of these data, packages can become complex and challenging to adapt to specialized needs. We therefore introduce ribofootPrinter, a suite of Python tools designed to offer an accessible and modifiable set of code for analysis of data from ribosome profiling and related types of small RNA sequencing experiments. Alignments are made to a simplified transcriptome to keep the code intuitive and multiple normalization options help facilitate interpretation of meta analysis, particularly outside coding regions. We demonstrate how mapping of short reads to the transcriptome increases the frequency of matches to multiple sites and we provide multimapper identifier files to highlight these regions. Overall, this tool has the capability to carry out sophisticated analysis while maintaining enough simplicity to make it readily understandable and adaptable.

## Introduction

**Figure 1.**
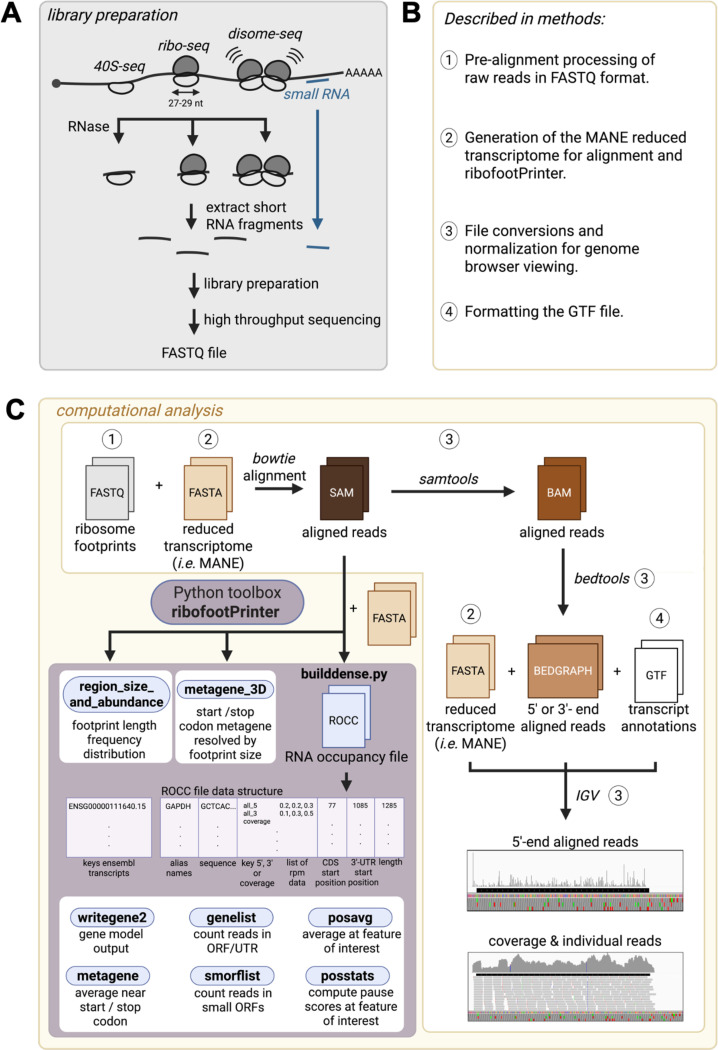
ribofootPrinter is a comprehensive python toolbox for analysis of short RNA datasets. **(A)** Schematic overview of short RNA datasets compatible with ribofootPrinter. Short RNA libraries are sequenced to generate high-throughput datasets in FASTQ format. **(B)** Additional information provided to enhance ribofootPrinter’s usability. **(C)** Steps to analyze data with ribofootPrinter (*left*). Ribosome profiling examples are provided throughout the manuscript. Pre-processed reads in FASTQ format are first aligned to the reduced transcriptome (FASTA) with bowtie and the resultant SAM file is processed into an RNA occupancy file (ROCC) using the *builddense* package. The ROCC file data structure contains ribosome occupancy data (5’- or 3’-assigned, or coverage) and sequence metadata. Data can then be analyzed with six downstream analysis tools: *writegene2, metagene, genelist, smorflist, posavg* and *posstats*. Both *region_size_and_abundance* and *3D_metagene* are compatible with SAM files and do not require conversion into a ROCC file. Steps to analyze data in the IGV browser (*right*). SAM files are converted into IGV-compatible BAM and BEDGRAPH files.

Ribosome profiling (also referred to as Ribo-seq) has become an established approach for revealing which genes are translated and how ribosomes are distributed along transcripts in cells (Ingolia et al. 2009; McGlincy and Ingolia 2017). Ribosome profiling data are generated by deep sequencing cDNA libraries that are generated from ribosome-protected mRNA footprints that typically measure ~28 nt in length (Figure 1A). However, in some experimental conditions, footprints can measure ~16 nt, ~21 nt, or >29 nt (Lareau et al. 2014; Schmidt et al. 2016; Guydosh and Green 2017; Wu et al. 2019; Ganesan et al. 2022) and reveal additional information. To analyze ribosome profiling datasets, reads are typically aligned to the transcriptome and downstream software packages can be used to reveal a rich and precise view of a cell’s translational program. Analysis can show the distribution and loading (translational efficiency) of ribosomes across messenger RNAs (mRNAs). It can also offer insight on where actively-translated open reading frames (ORFs) exist, including within non-canonical regions, such as 5’ and 3’ untranslated regions (UTRs) and out-of-frame sequences within coding sequences (CDSs) (Mudge et al. 2022). Related approaches (Dunn and Weissman 2016; Liu et al. 2020; Tjeldnes et al. 2021) for footprinting disomes (disome-seq) or 40S ribosomal subunits (TCP-seq) (Archer et al. 2016; Bohlen et al. 2020; Meydan and Guydosh 2020; Young et al. 2021) have enhanced the approach to address questions related to ribosome collisions and translation initiation.

Many capable software tools have emerged to analyze ribosome profiling data, in part because no single approach tends to fit every need (Limbu et al. 2024). However, one of the challenges faced by all approaches is how to handle the complex nature of mRNA splicing patterns in the cell. In humans, most transcripts are spliced into multiple isoforms, and this creates the problem of how to represent a particular gene’s level of ribosome occupancy, particularly when different isoforms (gene models) are translated with varied efficiency. Several solutions have emerged, including use of masking functions to exclude transcript regions where splicing patterns are variable (Dunn and Weissman 2016). Other approaches simplify the problem by mapping to the longest isoform or isoform with the most exons (Ishimura et al. 2014; Sendoel et al. 2017; Ahmed et al. 2019). Another challenge posed by splicing is the computational burden of converting between genomic and transcriptomic coordinate systems (Tjeldnes et al. 2021). This complexity also tends to make the code difficult to understand, raising barriers to code modification for beginners. Recently, the Matched Annotation from the NCBI and EMBL-EBI (MANE) project offered a curated transcriptome for *H. sapiens* with a single recognized (most likely to be used) isoform per gene (Morales et al. 2022).This unique resource offers a simple way to solve the problems associated with splicing for users who are not particularly concerned with unique isoforms.

At the alignment step, another challenge is deciding how to assign reads that can map to multiple locations in the transcriptome. While some allowance for multiple mapping locations is typical in bioinformatic pipelines, there is a limit to it and becomes particularly important when reads are short. As a result, it is important to use caution when interpreting data that map to transcriptome regions with high degeneracy. Once ribosome profiling data are aligned, many forms of analysis are typically carried out. One of the most common tasks is to create a “meta” plot by averaging ribosome profiling data over particular sequence motifs of interest (*i.e.* metagene or metacodon analysis). The simplest form of this analysis involves computing the arithmetic mean (or median) for read counts at every nucleotide position surrounding the feature of interest. However, the downside of this approach is that every feature (i.e. gene or codon) is not weighted equally in the average; highly expressed features tend to dominate. The need to consider alternatives to normalization is particularly critical when considering translation outside conventional coding sequences (Chen et al. 2020; Karasik et al. 2021; Mudge et al. 2022) . In such cases, the most appropriate method of normalization may not be immediately obvious, and it is therefore advantageous to consider multiple forms of normalization.

A final challenge of many computational tools is the loss of support over time and their eventual deprecation as changes are inevitably made to supporting packages and the programming languages themselves. This problem is particularly acute for packages that are complex and rely on extensive outside software to function. Therefore, simple and readily modifiable tools are advantageous for promoting longevity and continuity.

We therefore introduce ribofootPrinter, a precision Python toolbox for analysis of ribosome profiling data from any organism. The package simplifies the splicing problem by relying on a spliced transcriptome (for example, provided by the MANE project) and offers several functions that can be readily modified. Importantly, the software also offers the option of multiple normalization approaches for meta-analysis and can be further expanded. The simplicity of the approach makes this package appealing for both introductory programmers as well as users interested in a straightforward platform. ribofootPrinter is particularly useful for understanding several topics of growing importance, such as finding small ORFs, assessing ribosome stalling at sequences of interest, and evaluating translation outside of a transcript’s annotated coding sequence (CDS), which we also here refer to as the main open reading frame (ORF). The tool is compatible with 80S conventional ribosome footprinting, as well as 40S and disome footprinting, and can be adapted to other forms of small RNA sequencing.

### Implementation

#### Pipeline overview

We provide a complete and comprehensive analysis pipeline (Figure 1) for datasets obtained from ribosome profiling and other methods that use Illumina sequencing, such as sequencing of miRNA, piRNA, or CLIP-derived RNA. The first step is to pre-process the high-throughput sequencing FASTQ data file, which contains the raw sequencing reads (Step 1 in Figure 1B, C). Often this includes steps such as read trimming, deduplication, and removal of contaminating (for example, rRNA) reads, described elsewhere (McGlincy and Ingolia 2017).

**Figure 2.**
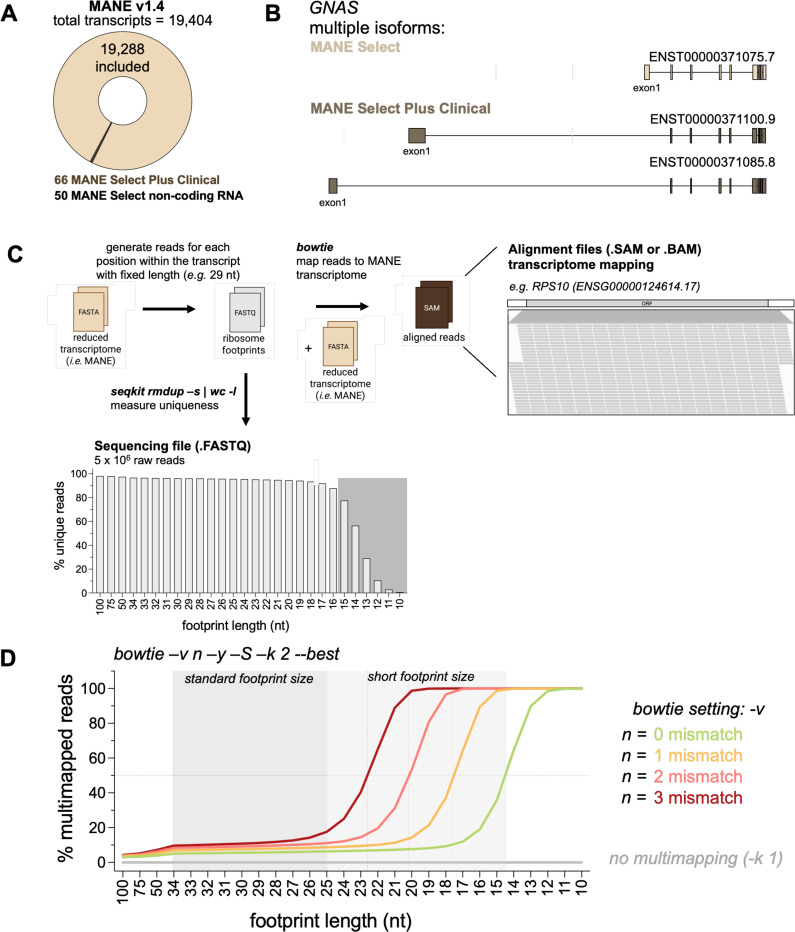
Tolerance to multimapping depends on read length and number of mismatches. **(A)** Overview of transcripts included in the MANE transcriptome in this study. Clinical and non-coding RNA sequences were excluded to reduce duplicate sequences. **(B)** Schematic representation of multiple isoforms of the *GNAS* transcript. The MANE select transcript isoform is shown in light brown and additional clinical select isoforms are shown in dark brown. **(C)** The reduced MANE v1.4 transcriptome was used to generate reads for each possible position within the transcript for a given length. These transcriptome-derived FASTQ files were aligned back to the exact same reduced transcriptome to obtain aligned SAM files. An example of the IGV browser is shown. Note the complete coverage of the entire RPS10 transcript. **(D)** Identification of multimapping events following alignment of transcriptome-derived FASTQ files against the reduced transcriptome using bowtie. Different *-v* settings allow for different number of mismatches during alignment. As expected, the FASTQ files containing shorter read lengths are more prone to multimapping while larger read lengths result in more uniquely mapped reads. Increasing the number of mismatches by changing setting (*-v*) result in increasing multimapped reads.

Next, to determine where reads map in the transcriptome, the processed FASTQ file must be aligned to the reduced transcriptome FASTA file (Figure 1C). We include instructions for using bowtie1 (Langmead et al. 2009), but other aligners can be used. Modification of the MANE (version 1.4) transcriptome to be compatible with ribofootPrinter is described in detail in the Methods (Step 2 in Figures 1B, C, and Supplementary Figure 1A). This transcriptome contains 19,404 transcripts but we excluded MANE Select non-coding RNAs to focus on protein coding transcripts (Figure 2A). In addition, we also excluded the MANE Select Plus Clinical set to avoid duplicate gene names and sequences. This results in a transcriptome with mostly unique transcripts that reduces multimapping (*i.e.* many sequences that a single read could be aligned to). For example, the *GNAS* gene (ENSG00000087460.29) has two isoforms that are annotated as MANE Select Plus Clinical and therefore eliminated in this process (Figure 2B). These removals leave a total of 19,288 transcripts for alignment (listed in shortnames.fasta, see Methods ).

The SAM file that is generated by the alignment can then be converted into a BED or BIGWIG file that is normalized by sequencing depth (*i.e.* units of RPM) for visualization in a genome browser, such as IGV (Thorvaldsdottir et al. 2013) (Step 3 in Figures 1B,C, and Supplementary Figure 1B). Unlike traditional approaches where RNA reads are aligned to the genome, this transcriptome alignment produces tracks that lack introns. Feature annotations, such as alternate names and ORF boundaries, are provided by creating of a GTF file that can be loaded by the browser (Step 4 in Figures 1B,C, and Supplementary Figure 1B). Additionally, the SAM file can be converted into a specialized RNA occupancy (“ROCC”) file that is used for downstream, in-depth analysis using ribofootPrinter (Figure 1C). The code is written in Python 3 and requires the BioPython package (Cock et al. 2009) for sequence manipulations. All functionalities typically run within a few minutes on modern personal computers.

While the simplification step of a reduced transcriptome represents an obvious compromise, it facilitates downstream analysis by allowing faster performance and eliminates artifacts associated with using the longest (but not most common) transcript isoform or eliminating common genes due to ambiguous splicing. This curation process also avoids artifactual peaks common in pseudogenes and non-coding RNAs. In addition, it allows easy visualization of translation events in genome browsers, such as IGV, without interference from introns.

#### Reduced transcriptome alignment considerations

To test basic properties of read alignment to the MANE 1.4 transcriptome, particularly the tendency to map to multiple locations, we generated a FASTQ file containing every possible read that could be derived from the transcriptome sequences (Figure 2C). Since ribosomes can protect a range of mRNA lengths, depending on preparation conditions, we generated files for footprint sizes ranging from 10–100 nt. These lengths also span the typical lengths of other small RNAs of interest, such as miRNAs and piRNAs. We found that 2% of the 100-mer reads were not unique (Figure 2C). This result could potentially be attributed to repetitive sequences, conservation of sequences between genes, different isoforms of the same gene, or overlapping transcripts (Veeramachaneni et al. 2004). As expected, the number of unique reads declined as read length (and therefore information content) was reduced and fell off steeply below about 15 nucleotides) (Figure 2C, fastq file cartoon gray background).

Next, we aligned these transcriptome-derived FASTQ files back to the transcriptome using bowtie (Figure 2C) to explore how settings in the alignment affect this process. We confirmed that 100% of the reads mapped following bowtie mapping using different settings. Consistent with our finding that shorter (particularly <15 nt) reads contain a large proportion of non-unique sequences (see Figure 2C), we found that the fraction of reads mapping to multiple locations increased for shorter read lengths (Figure 2D). To maximize the number of reads that mapped uniquely, we specified 0 mismatches in the alignment (*-v 0*) to ensure perfect alignment of the read to the transcriptome. With this setting, we found 65% of the 15-mer reads mapped uniquely (Figure 2D) while, in contrast, 90% of 20-mer reads mapped uniquely. We noted this trend changed as the number of mismatches was relaxed, going from 8% with 0 mismatches, 14% with 1 mismatch, 53% with 2 mismatches and 99% with 3 mismatches for the 20-mer reads (Figure 2D). Another consideration is that since the transcriptome only contains positive-sense annotated transcripts, running alignments that disallow mapping to the reverse complement strand *(--norc*; no reverse complement) enhanced performance (6 vs 8% multimapping with 0 mismatches for 20-mer reads). Whether limiting antisense mapping is advantageous may depend on the particular conditions of the experiment. For example, a tendency for some bidirectionality in many promoters may contribute to antisense reads in real-world datasets (Nemsick and Hansen 2024).

To help characterize the inherent repetitiveness of the transcriptome that underlies the tendency of reads to map to multiple locations, we generated multimapper identifier files (*mm_id.bedgraph)* for a range of read lengths to highlight these regions in a genome browser (Supplementary Figure 2). The metric encoded in these files measures the tendency for a read to map to multiple locations with higher values indicating more multimapping (see Methods ).

**Figure 3.**
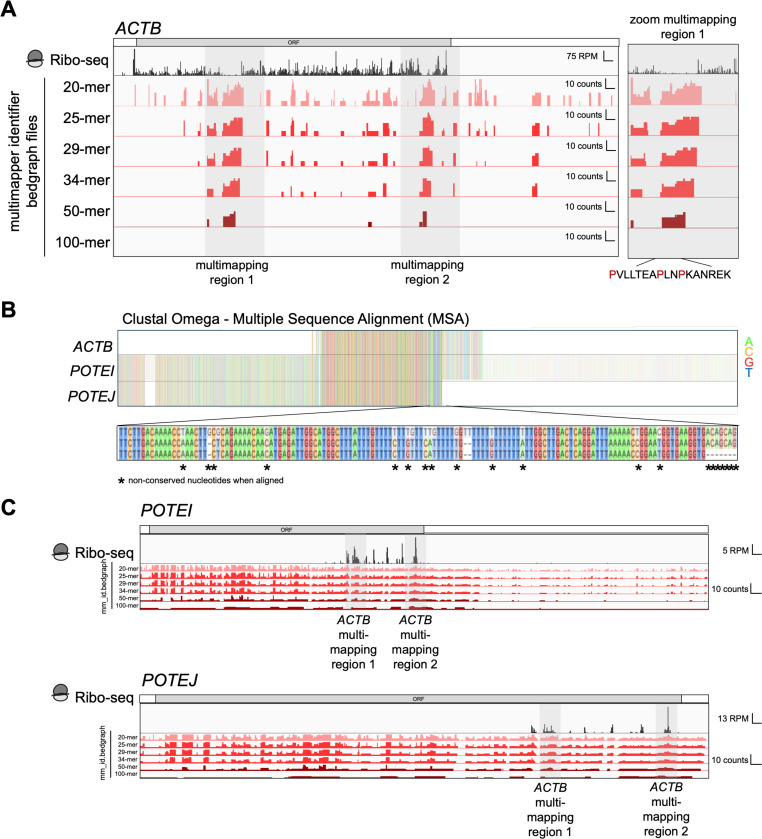
Multimapper identifier file identify multimapping events in *ACTB*. **(A)** IGV browser view of the *ACTB* transcript (encoding β-actin) with multimapper identifier files (mm_id) loaded in different shades of red. Multimapper identifier files containing larger sequence lengths are less likely to multimap (show in darker shades of red) while shorter sequences lengths are more likely to multimap (shown in lighter shades of red). Regions which are identified as likely to be non-unique (*i.e.* multimapping regions) are highlighted with a grey box. *ACTB* contains two strong multimapping regions. The multimapped regions correlate with a decrease in footprints in the ribo-seq dataset caused by default bowtie settings for alignment which only report one single best alignment in case of multimapped reads. The zoomed box of multimapped region 1 highlights the presence of prolines which are ribosome-stalling inducing sequences. This could lead to an underestimation of stalling events. **(B)** Clustal Omega alignment of transcripts *ACTB, POTEI* and *POTEJ* reveal strong sequence similarities leading to multimapping. The sequences between *ACTB* and *POTEI POTEJ* are not 100% identical consistent with absence of multimapped regions in the 100-mer multimapper identifier file in **A**. **(C)** IGV browser view of *POTEI* and *POTEJ* transcripts. The multimapping regions identified in *ACTB* in **A** are highlighted in grey boxes and reveal the footprints that are likely derived from *ACTB*. Besides the multimapped regions, very few footprints are observed within the ORF indicating that these transcripts are not translated.

To demonstrate the utility of the multimapper identifier files, we examined the *ACTB* transcript (encoding β-actin). We noted several regions with a high level of multiply mapping reads (Figure 3A, highlighted in grey). We identified two transcripts, *POTEI* and *POTEJ*, that contain a high level of sequence conservation with *ACTB* and are responsible for the non-unique mapping (Figure 3B) (Madeira et al. 2024). Consistent with the analysis above, the tracks made for longer read lengths contain lower values compared to those made for shorter reads. When we examined our Ribo-Seq alignment where limited multimapping was tolerated (multi-mapping reads assigned to one location at random, bowtie setting *-k 1*), we noted a decrease in ribosome footprints within these regions (Figure 3A, zoom). We further noted that the *POTEI* and *POTEJ* transcripts generally had no footprints mapping to them except for the multimapped region (Figure 3C). We therefore conclude that reads from these regions likely derive from *ACTB* and are evenly divided between three transcripts, resulting in diminished mapping to *ACTB* and unexpected mapping to *POTEI* and *POTEJ*. As a result, performing a ribofootPrinter’s pause score analysis (see later) on this region for proline, which is known to induce ribosome pausing (Schuller et al. 2017), would tend to underestimate the pause score (Figure 3A, zoom). While the examples of non-unique sequences within *ACTB* arise from small regions of homology, we note other examples where entire exons are conserved between many genes in the MANE transcriptome. An example is the protocadherin protein family that is annotated with the *PCDHG* prefix. The multi-mapping problem has been long established in the bioinformatics field and best approach for handling it (allowing 0, 1, or many locations to be mapped, or adding additional processing) will vary according to the needs of particular applications (Wang et al. 2016; Halpin et al. 2020). Our analysis here is aimed at making these limitations clear as a helpful resource for users.

#### Distribution of footprint lengths

**Figure 4.**
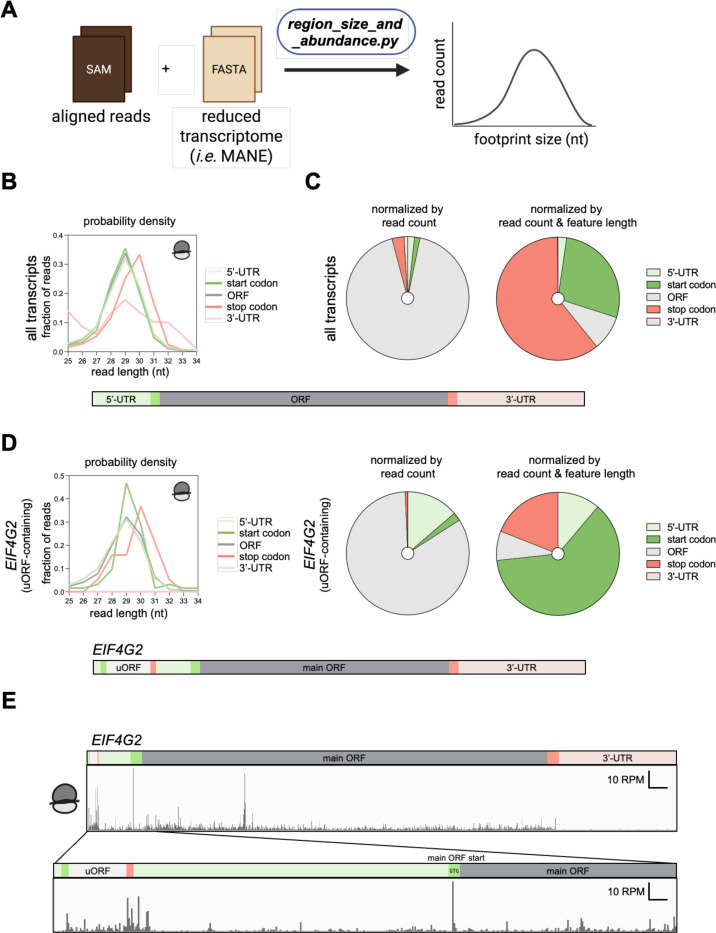
ribofootPrinter analysis to determine read length distribution within UTRs, ORF, start or stop codon can be used to identify uORF containing transcripts. **(A)** Schematic representation of the *region_size_and_abundance* package. This package uses a SAM file as input. **(B)** The distribution of footprints within UTRs, ORF, start or stop codons for all transcripts normalized by probability density. This allows direct comparisons between different features revealing longer stop reads in our dataset. **(C)** Abundance of reads across the transcriptome. The pie chart on the left gives the percentage of total reads that map to each region. The pie chart on the right gives the percentage of reads normalized by the length of the transcript to which they map. The start and stop codon regions are most abundant since they are short and contain a large proportion of reads. **(D)** Analysis of footprint distribution for uORF containing transcript *EIF4G2* reveals a higher number of reads in the 5’-UTR, as expected. **(E)** IGV browser view of the uORF containing *EIF4G2* transcript confirms a uORF seen by footprints between start and stop within the 5’-UTR.

Following alignment of the ribosome footprints to the reduced MANE transcriptome (see Figure 1C), the SAM files containing mapped reads can be used to determine the distribution of read lengths (Figure 4A). This analysis can be helpful in determining a dataset’s quality, for example revealing cases where the RNase treatment was insufficient to fully trim footprints to 28 nt. In addition, this function can also be used to reveal different species in an experiment, for example short-read ribosome profiling, disome or trisome profiling, and 40S profiling methods. The package also calculates the abundance of footprints according to the sequence region to which they map (*i.e.* start and stop codon, main ORF, and UTRs). This is done by defining an adjustable window around the start and stop codon and otherwise assigning reads to the main ORF or a UTR region (Supplementary Figure 3 top). The distribution of read lengths can be computed from these region-based assignments. Plotting the data in this way (Figure 4B) reveals that ribosome-protected footprints at the stop codon are consistently 1 nt longer compared to other regions, as reported previously (Ingolia et al. 2011). While the read length distributions can be plotted as a standard probability density (as shown in Figure 4B), other normalization options are available. The most straightforward option is to report raw reads or reads normalized by library depth (RPM units) to facilitate comparison between samples. This approach generates an abundance distribution (Figure 4C, left) that shows most reads within the coding region (main ORF). An alternative way to represent abundance is to compute the fraction of the read density per unit length for each read. In this way, regions that are short but densely populated with reads, will receive higher representation (Figure 4C, right). This approach shows start and stop regions to be densely enriched due to a peak on those codons, while the UTRs are lowly represented due to the low mapping of reads per length in these regions. If desired, the length distribution can be combined with abundance normalization to simultaneously visualize both outputs in a single plot (Supplementary Figure 3 bottom). In addition, a subset or single transcript can be studied by providing a list containing gene names of interest (in ENSG format). For example, we determined the read length distribution and region abundances of the uORF-containing *EIF4G2* transcript (Figure 4D, left, ENSG00000110321.19). The abundance plots (Figure 4D center and right) show more reads in the 5’ UTR than the total transcriptome (Figure 4C). This is consistent with the IGV track of *EIF4G2* where footprints exist within the uORF region (which is annotated as 5’ UTR) (Figure 4E). The histogram of read lengths is similar, as expected (Figure 4D, right).

#### Generation of the RNA occupancy files (ROCC)

**Figure 5.**
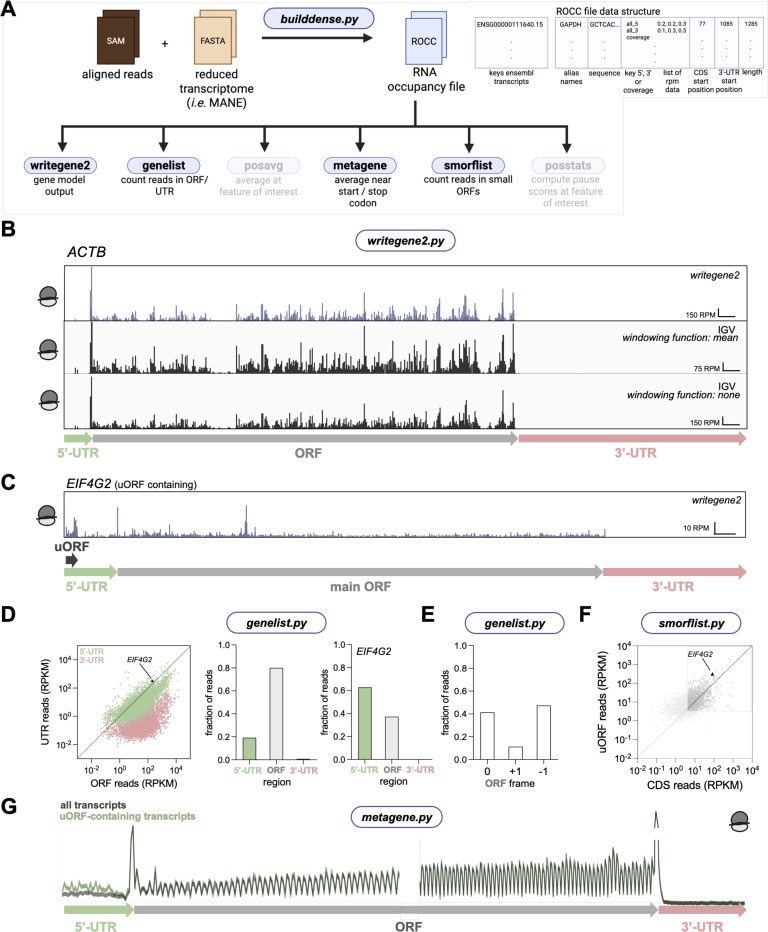
ribofootPrinter analysis at the transcript level can be used to identify translation outside the main ORF. **(A)** Schematic representation of the *writegene2, genelist, metagene* and *smorflist* packages. These packages use a ROCC file as input. **(B)** Example output of mapped ribosome profiling reads on a gene model for *ACTB* generated by *writegene2* (shown in purple). The data are compared against IGV browser views and different windowing settings which influences the representation of the data (shown in black). **(C)** Example output of mapped ribosome profiling reads on the uORF-containing *EIF4G2* transcript generated by *writegene2*. **(D)** Output of *genelist* shows that 5’-UTRs typically have more reads than 3’-UTRs (each dot is a single individual transcript). uORF-containing transcript *EIF4G2* is highlighted as it contains more 5’-UTR reads compared to ORF reads (*left*). Analysis of mRNA regions reveals the vast majority of reads map to the CDS with a low-level mapping to the 5’-UTR and even less to the 3’-UTR (*center*). Analysis of the uORF containing *EIF4G2* transcripts reveals a large number of 5’-UTR reads, as expected and consistent with C. **(E)** The *genelist* package also identifies the reading frame of the end of mapped reads (*center*). **(F)** Result of small ORF analysis using *smorflist* (each dot is a single predicted uORF) in 5’-UTRs reveals that, as a function of CDS reads, density of reads on individual uORFs is comparable to the CDS (dots more or less along diagonal). This analysis of actual uORFs contrasts with the result in **(D)** where analysis of entire 5’-UTRs reduces the apparent density due to inclusion of regions without uORFs (data further above diagonal in **F** vs **D**). **(G)** Metagene plot for start and stop codons reveals 3-nt periodicity in the coding sequence. It also shows low level translation in the 5’-UTR and nearly no translation in the 3’-UTR, consistent with **D**. Predicted uORF-containing transcripts are overlayed and show increased translation in the 5’-UTR, as expected. Note that 5’ ends are mapped in **B-G**.

Following bowtie alignment, a central step in the ribofootPrinter pipeline is to create data structures (ROCC files) to hold the RNA occupancy data from SAM files by using the *builddense* package (Figure 5A). These structures are created using python dictionaries, with keys for each gene, to include both information, such as gene name, sequence, UTR positions, and normalized (rpm) footprint data for a size range of interest (i.e. 25–34 nt). In this way, these occupancy files keep metadata and experimental data together. As with other tools (Dunn and Weissman 2016), information is saved for both 5’ and 3’ end assignment of mapped reads, as well as the option to output the coverage of the gene (*i.e.* the entire read). We also provide an option for mapping of paired-end bowtie mapped reads. As shown previously, 3’ end assignment often offers superior reading frame alignment for ~16-nt or ~28-nt reads from ribosomes in yeast cells, while 5’ end alignment is superior for 40S subunits in yeast or 80S ribosomes in many human cell types (Guydosh and Green 2017; Karasik et al. 2021; Young et al. 2021).

#### Individual transcript analysis

With these RNA occupancy (ROCC) files in hand, several tools are available for analysis (Figure 5A). One of the most basic applications is to output read occupancies as a function of nucleotide position for a particular gene model. This is accomplished with ribofootPrinter’s *writegene2* package. The output data in RPM is provided in csv format which can then be used to create images of ribosome occupancy on a spliced transcript. Like the spliced transcript files created for use in IGV (BED or BW, described above in Figure 2D) and other transcript-centric viewers (Kiniry et al. 2019), this output offers the advantage over traditional genome viewers in that it excludes intronic regions (Figure 5B, top, example of *ACTB* gene model). However, ribofootPrinter’s *writegene2* offers an advantage of avoiding artifacts due to averaging performed by IGV’s default windowing function. Comparison between the gene model for *ACTB* produced by *writegene2* against the IGV track shown above revealed peak height differences when using IGV’s default *mean windowing function* setting (Figure 5B, center). If the transcript is too long to display a single peak for each position, this setting causes all peaks within a certain window to be averaged, presumably as a way to improve performance. While the *windowing function* in IGV can be changed to *none* (Figure 5B, bottom), this setting introduces different artifacts by displaying downsampled data. Overall, ribofootPrinter’s *writegene2* reliably outputs the RPM for each position of the transcript without artifacts that are often introduced by genome browsers.

RibofootPrinter’s *genelist* package includes the ability to quantitate total read counts per transcript, reported as both raw reads for differential expression analysis tools, such as DESeq2 (Love et al. 2014), or normalized to total mapped reads and length (in kilobases) of gene models (RPKM) (Figure 5A). Outputs are offered for both coding sequence (ORF) and UTR regions to give a quick portrait of the level of 5’ and 3’-UTR translation levels, values that are known to change due to alterations in the fidelity of initiation or ribosome recycling, respectively. For example, since *EIF4G2* contains a uORF, it is expected to contain a larger number of reads in the 5’-UTR compared to a uORF-less transcript (Figure 5C, Figure 4D, E). Globally, our riboseq sample data reveal that footprint levels in 5’-UTRs are considerably greater than in 3’-UTRs as is expected, since under normal conditions more uORF translation occurs compared to stop codon readthrough and consequent 3’-UTR translation (Figure 5D) (Mudge et al. 2022). As expected, *EIF4G2* exhibited a higher proportion of 5’-UTR reads compared to the entire population. In addition, an optional mode outputs reads in each of the 3 reading frames of the coding sequence, allowing visualization of possible frame defects that can result from events such as frameshifting or leaky scanning (Figure 5E). Note that use of this feature assumes the footprint length distribution is narrow enough to distinguish between frames. Factors such as insufficient or inconsistent RNase treatment across samples can limit the utility of this analysis. In general, which frame is considered “0” relates to the “shift” value that estimates the distance between the 5’ or 3’ end of the read and internal features, such as the ribosome A site (see section below on the *metagene* function for further discussion).

#### Small ORF analysis

Accurate quantitation of ribosome footprints on small open reading frames outside coding sequences represents an active area of investigation. These upstream open reading frames (uORFs) in 5’-UTRs and downstream open reading frames (dORFs) in 3’-UTRs are emerging as important regulatory elements that can either enhance or inhibit translation of the main coding sequence (Lin et al. 2019; Hinnebusch et al. 2016). While metagene analysis (see below) and total quantitation of reads across a UTR region can offer some insight about translation in UTRs, precision estimation of a particular small ORF’s usage is more complicated since small ORFs tend to be overlapping and may initiate with multiple start codons. In addition, initiation may occur with a near-cognate start codon (1-bp mismatch from ATG). In ribofootPrinter’s *smorflist* package (Figure 5A), quantitation of reads on small ORFs is implemented by counting in-frame reads only on all potential small ORFs, thereby excluding most reads from overlapping out-of-frame ORFs. The small ORFs that are analyzed can be filtered by start codon (perfect match or 1-bp mismatch) and by length, since longer ORFs will be less subject to noise. The output of this analysis can then be combined with appropriate thresholding to identify likely expressed small ORFs. In a sample dataset, ribosome density across uORFs reveals many uORFs with expression levels comparable to that of the main ORF, including that within *EIF4G2* (Figure 5F).

#### Metagene analysis

The ROCC file offers the ability to perform average (*metagene* function) analysis at start or stop codons of coding sequences (Figure 5A). The package calculates the average read count at a range of nucleotide positions across transcripts that are aligned by their start or stop codon (Supplementary Figure 4A). This analysis at start codons is essential to estimate the position of the ribosome P site with respect to either the 5’ or 3’ end of mapped footprints. This “shift” value is then used by other analysis tools in the package. Typically, 5’ assigned ribosome profiling in either yeast or human cells results in footprint peaks 12 nt upstream of start codons, showing that the first base of the P site is about 12 nt away from the 5’ end (Supplementary Figure 4B).

In addition, metagene analysis for riboseq data also offers a visual metric of reading frame fidelity by revealing how strong the periodicity is in the coding region (Figure 5G). As noted above, this periodicity is also a function of factors that affect the read length distribution, such as RNase treatment. It can also visually reveal the level of translation in UTRs and directly show how it compares with the main ORF. Comparison of a metagene computed solely from the previously identified uORF-containing transcripts in Figure 5F (using an available subsetting function) clearly reveals higher levels of translation within the 5’-UTR compared to the main ORF (Figure 5G). Traditional metagene analysis is performed using an arithmetic mean to compute the average (setting: *equalweighting 0*, units of rpm). However, this gives more weight to the most highly expressed genes in the transcriptome and can result in artifacts. Therefore, an alternative with equal weighting of all genes is made available (setting: *equalweighting 1*, units of fraction of reads). Weighting is performed by counting total reads within the coding sequence of the gene, subject to thresholding options, and normalizing the data to this prior to averaging. Note that this setting is not recommended for use with more specialized analysis, such as 40S profiling datasets where the coding sequence cannot be used to evaluate expression level.

#### Metasequence analysis and pause scores

**Figure 6.**
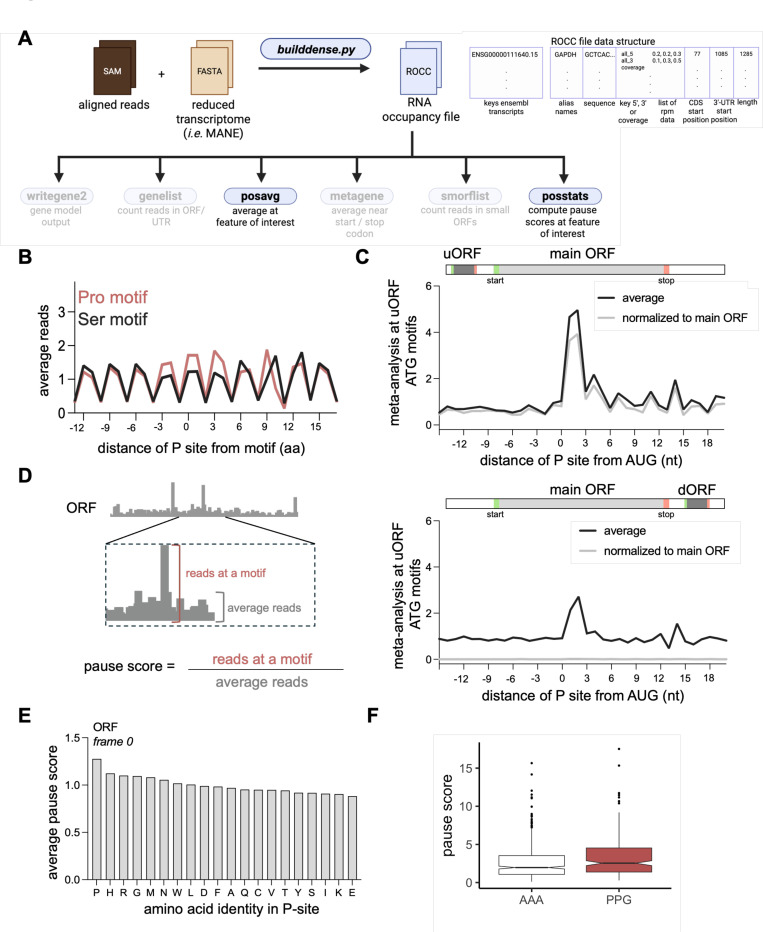
ribofootPrinter analysis to analyze pause scores of riboseq data. **(A)** Schematic representation of the *posavg* and *posstats* packages. These packages use a ROCC file as input. **(B)** Average analysis using *posavg* of reads at proline (Pro) and serine (Ser) codon motifs reveals a peak when the Pro motif (but not the Ser motif) is placed in the P site. **(C)** Metaposition analysis of AUG codons in the 5’-UTR (*top*) reveals a clear peak and increased ribosome occupancy and 3-nt periodicity after the peak. This result is consistent with efficient translation of uORFs. Whether data is normalized to a local window (black) or to the respective ORF sequence (grey) does not dramatically affect the result since uORF translation levels are comparable to main ORF translation levels. Analysis of AUG codons in 3’-UTRs (*bottom*) shows a similar peak when reads are combined with a locally-normalized average, showing dORF translation does take place. However, the lack of a peak when reads are normalized to the ORF (grey) shows the translation level is quite low. **(D)** Schematic representation of pause score calculations. **(E)** Pause scores from positional average analysis at every amino acid reveals Pro induces the most stalling. **(F)** Comparable analysis of the distribution of all pause scores at AAA and PPG tri-amino acid motifs shows a clear difference in the overall distributions with more pausing on PPG.

Computing the average read level around sequence-specific positions within gene models (“metasequence” or “metacodon” analysis) is critical for estimating levels of ribosome stalling during elongation. It can also reveal usage of small ORFs in UTRs (uORFs and dORFs) or internal to coding sequences (iORFs). To accomplish this, ribofootPrinter’s *possavg* package accepts a specific nucleotide or amino-acid sequence and then computes average ribosome footprints within a defined window around each occurrence of these motifs in the transcriptome (Figure 6A). Averages are subject to thresholding over the window of interest to eliminate low-coverage artifacts. This analysis can be used to check particular motifs for tendency to slow elongation in coding sequences. For example, when footprints are averaged when in-frame Pro vs Ser codons are in the P site, Pro clearly slows the ribosome (Figure 6B) more than Ser, as is expected (Schuller et al. 2017).

Metasequence analysis at start (AUG) codons within UTR regions offers a way to check for ORF translation. Critically, the average can be locally normalized as a probability density within the window around the motif or normalized to the gene model’s respective coding sequence. For analysis of uORFs, the outcome is similar with both methods since ribosome occupancy on uORFs is often comparable to that of coding sequences (Figure 6C, top). In contrast, ribosome occupancy on dORFs is quite low compared with coding sequences and therefore the locally normalized analysis clearly reveals translation on dORFs while the CDS-normalized analysis only shows a very small level of translation (Figure 6C, bottom). In addition, the code can also compute a pause score from the meta-analysis plots. The score is computed by dividing the counts in the peak by the background level (Figure 6D). Analysis for every single amino acid in the ribosome P site clearly shows how Pro is the slowest amino acid (Figure 6E), as expected.

Finally, to gain insight about the distribution of pause scores, pause scores at individual mRNA positions can be computed, subject to thresholding of background levels. This analysis enables more advanced statistical analysis of the distribution of pause scores at motifs of interest and can show global trends. For example, it reveals that translation of Pro-Pro-Gly is slower than Ala-Ala-Ala globally (Figure 6F).

#### 3D metagene analysis

**Figure 7.**
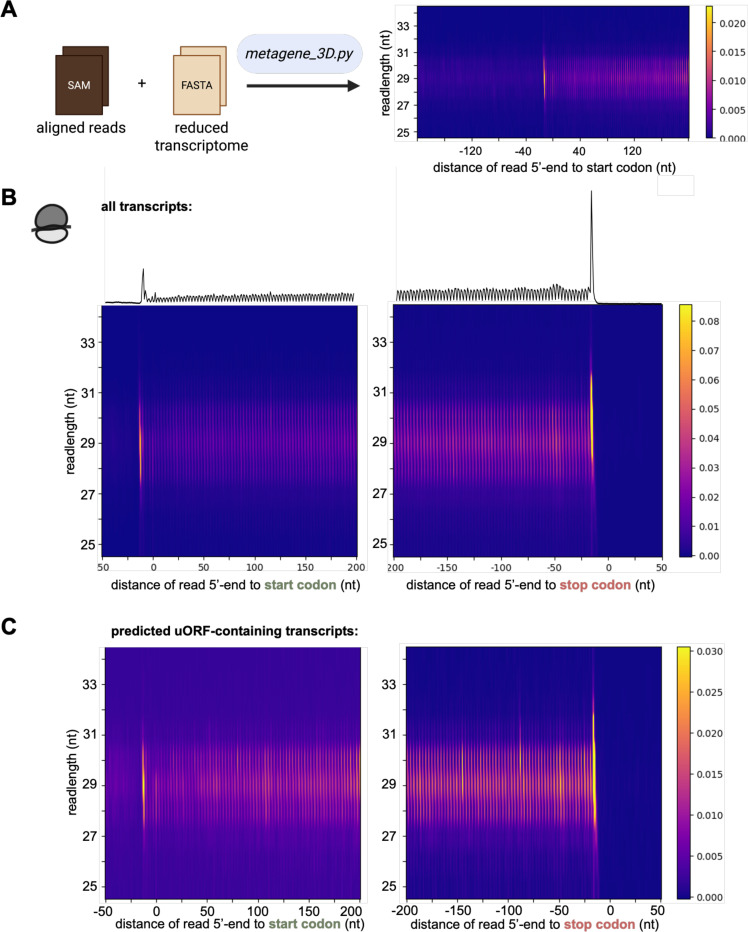
3D metagene analysis provides additional information on read length. **(A)** Schematic representation of the *metagene_3D* package. This package uses a SAM file as input. **(B,C)** Comparison of the 3D metagene around start and stop codons for all transcripts **(B)** and predicted uORF-containing transcripts **(C)** reveal additional signal within the 5’-UTR when uORFs are present.

The ribofootPrinter package can also carry out metagene analysis at start or stop codons as a function of read length. The 3-dimensional (3D) metagene plot that is created by this analysis reveals how different species in a population (*i.e.* ribosomes protecting different footprint sizes) are distributed across a transcript. When performed for both 5’ and 3’ assignment, these plots can reveal how a given ribosome footprint straddles particular features, such as the start codon. Unlike a regular metagene plot, this tool works directly on SAM files, rather than ROCC files, due to the large amount of data required (Figure 7A). Similar to a conventional metagene plot, the location of the mapped read, around start or stop codon, is plotted on the x-axis. The footprint size is then plotted on the y-axis and the read abundance is shown as a heatmap (high abundance in yellow, low abundance in dark purple), thus using all 3 dimensions. It also outputs a matching conventional metagene plot.

In the 5’-assigned 3D metagene plot for our riboseq dataset, we observed that the ribosomes that accumulate on start and stop codons in a conventional metagene (Figure 7B, *top* trace) are also discernable in the 3D metagene plot as yellow regions (Figure 7B), consistent with the ribosome footprint abundance analysis (see Figure 4C). In addition, we found a higher abundance of ribosomes about 10 codons upstream of the stop codon. These peaks are likely formed by ribosomes that stall behind the ribosome at the stop codon and are therefore consistent with disomes forming at this position. We also generated a 3D metagene plot for the previously identified uORF-containing transcripts in Figure 5F (using an available subsetting function) and observe an increased ribosome occupancy within the 5’-UTR specifically, as expected (Figure 7C).

#### Other profiling methods

**Figure 8.**
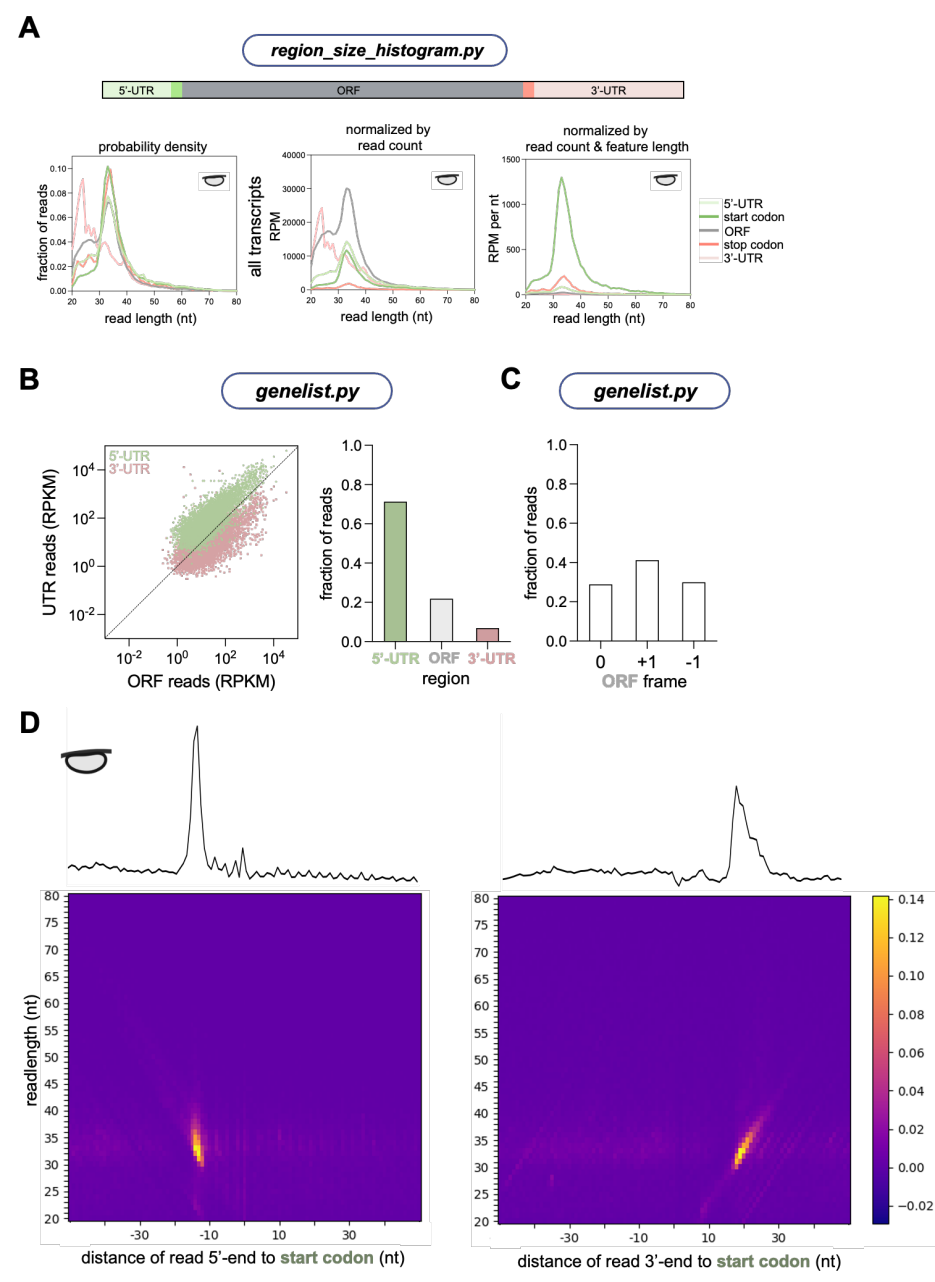
ribofootPrinter is compatible with 40S profiling and other datasets. **(A)** Read length distributions were computed with *region_size_abundance* and scaled using abundance outputs. On the left, the probability density is shown for each region. In the center, the scaling is normalized by overall read abundance in each region. On the right, the histograms are scaled by the abundance normalized to the length of the gene mapped to by each read. **(B)** Output of *genelist* plotted at the level of gene models shows that, as expected, 5’-UTRs of 40S profiling datasets typically have more reads than the ORF and 3’-UTR (each dot is a single individual transcript) (*left*). Analysis of mRNA regions reveals the vast majority of reads map to the 5’-UTR with a low-level mapping to the main ORF and even less to the 3’-UTR (*right*). **(C)** The *genelist* package identifies no specific reading frame preference for 40S footprint ends, as expected. **(D)** Metagene and 3D metagene analysis of 40S profiling data at start codons. This reveals that 40S subunits are mostly on start codons. Note the wide footprint length distribution protected by 40S subunits at the start codon is likely caused by presence of initiation factors. Comparison between 5’ and 3’ aligned plots (left vs right) shows that the 3’ ends of reads vary with read length since peak is less vertical (more diagonal) than for 5’ reads on left.

As noted above, Ribofootprinter is compatible with other types of ribosome profiling preparation methods and other types of short-read sequencing. We therefore examined the performance of the package with 40S subunit profiling (TIS-seq or 40S profiling). First, we determined the footprint length distribution as a probability density (Figure 8A left) and then normalized it according to read count, and read count and feature length (Figure 8A center/right). Unlike the 80S footprinting data, 40S footprints protect a wider range of sizes with a peak around 32 nt and tails extending to both shorter and longer footprints. Similar to our 80S dataset, we found that abundance computed by read depth reports the large absolute number of footprints within the ORF (Figure 8A center). Abundance normalized by feature length, in contrast, shows how the density of 40S subunits per unit mRNA length is greatest at start and stop codons, followed by the 5’-UTR (Figure 8A, right). This suggests 5’-UTR scanning is relatively quick and that 60S association and removal are likely slower.

Next, we investigated our 40S dataset using the *genelist* package. As expected and consistent with our observations above, we found that the majority of the 40S footprints are located within the 5’-UTR (including start codon) (Figure 8B). In addition, the frame distribution of ORF 40S footprints is nearly random (Figure 8C). This indicates that a the 40S footprints within the ORF likely result from a process that is not dependent on reading frame, such as scanning. If the footprints resulted from translating ribosomes that had fallen apart during sample preparation, the frame distribution would be less equally represented between the three different frames. as shown for 80S data in Figure 5E.

In addition, we plotted the conventional and 3D metagenes for the 40S profiling dataset at start codons (Figure 8D). Since 40S profiling captures pre-initiation complexes, the majority of the footprints are expected to be localized within the 5’-UTR and start codon. Consistent with this prediction, we find that most of the initiating 40Ss are present on the start codon (likely awaiting recruitment of the 60S subunit) and within the 5’-UTR (likely scanning for a start codon), as shown previously (Bohlen et al. 2020). Comparison of the 5’- and 3’-assigned plots shows that the longer footprints are also enriched at the start codon (>40 nt) and tend to be extended on their 3’-end.

#### Considerations for preparation of other transcriptomes

The MANE reduced transcriptome contains a carefully curated set of coding transcripts containing a single isoform that represents the most biologically relevant transcript (Morales et al. 2022). Currently, mitochondrial genes are not included in the MANE datasets. In addition, curation of coding transcripts was only completed for human. If desired, a custom transcriptome can be generated for other species, mitochondria, viruses, and others to be fully compatible with ribofootPrinter. To generate a custom transcriptome, both sequence information and metadata, such as ORF boundaries and transcript length, need to be obtained to generate the FASTA file required for ribofootPrinter (see Methods ). For example, a fully annotated genome of respiratory syncytial virus (RSV) is available from NCBI (nucleotide: KT992094.1). Both sequences for the full transcripts and annotated CDS can be downloaded and are sufficient to create the FASTA file.

If these transcriptomes will be used in addition to the human transcriptome for example, it is advisable to generate multimapper identifier files for both transcriptomes to identify any similarities between two transcriptomes that lead to ambiguous mapping.

## Conclusions

We have introduced ribofootPrinter, a readily configurable software toolbox for the analysis of ribosome profiling data. Its approach of reducing mapping complexity by using a reduced transcriptome represents a practical solution to the challenge of aligning reads. This compromise then enables the use of and high-resolution tools for analysis of translation, making it a powerful and easy-to-use resource for researchers in the field.

To address the problem of unique mapping inherent in all short-read alignments (Deschamps-Francoeur et al. 2020; Almeida da Paz et al. 2024), we describe multimapping identifier files to view alongside mapped data in a genome bowser to highlight regions transcriptome regions that are prone to multimapping. In addition, ribofootPrinter’s multiple options for meta analysis normalization offers multiple solutions to a problem where needs differ between applications. While no package serves the need of every user, we believe ribofootPrinter offers a useful balance of simplicity and key functionalities that will make it a good choice for many users. In addition, it offers a simple way to get ribosome profiling footprint occupancy and respective mRNA transcript sequences into standard Python list and string data types to help facilitate the development of new forms of analysis.

We also look forward to future enhancements to improve the capabilities of the software. For example, we look forward to adding reduced transcriptomes for other species. It would also be desirable to include capabilities for finding pause peaks and report the underlying sequences as potential pause sites. In addition, more sophisticated data structures could allow for the storage of variable read lengths to facilitate more precise analysis of read-length specific mapping of ribosome footprints. While including data for every size tends to becomes overwhelming for data storage, alternative binary approaches could resolve this (Ozadam et al. 2020). We also anticipate that the growing understanding that translation outside of coding regions is important for regulating gene expression and generating functional peptides will necessitate additional tools for characterization of ORFs in noncoding regions (Chen et al. 2020). Finally, we expect additional tools could be created for analysis of other types of small RNAs besides ribosome footprints, such as those generated by clip-based approaches. We believe the straightforward design of this ribofootPrinter will enable continued growth and adaptation to study these phenomena.

## Methods

ribofootPrinter is available on Github: https://github.com/guydoshlab/ribofootPrinter2.0-beta.

### ribofootPrinter compatibility and packages

The software is best run inside a Python 3 virtual environment (venv) where Biopython (and other required packages, which include matplotlib, pandas, openpyxl) have been installed. We assume the user has appropriately pre-processed ribosome footprint reads, including steps to trim linkers, remove duplicates using unique molecular identifiers (UMIs), and subtract rRNA and other non-coding RNA sequences. At this stage, the reads can be aligned to the reduced transcriptome of choice using bowtie. In this study, the reduced MANE v1.4 transcriptome (shortnames) FASTA file was used and can be downloaded from the Github page or created using the instructions provided therein.

Once SAM files are obtained from the bowtie alignment, they can be converted into the stored read data structures (ROCC files) using the *builddense* function (described below). ROCC files contain important mapping information and serve as input files for *writegene2, metagene, genelist, smorflist, posavg* and *posstats* packages. The packages *region_size_and_abundance* and *metagene_3D* are run directly on the SAM file.

The longnames.fasta file used for running ribofootPrinter can be downloaded from the Github page. The longnames.fasta file must adhere to the following header format with all fields separated by “|” characters, as shown in this example:

>ENSG00000111640.15|ENST00000229239.10|ENSP00000229239.5|NM_0020

46.7|NP_002037.2|GAPDH|1285|UTR5:1–76|CDS:77–1084|UTR3:1085–1285

The first position should be the formal gene name, i.e. “ENSG00000111640.15” followed by the alias gene name in the sixth position. This is followed by overall gene length. The final 3 positions should include the prefixes “UTR5:”, “CDS:”, and “UTR3:” followed by the start and end position of that region, separated by a hyphen.

All analysis routines are performed on the command line by passing relevant parameters to the Python script. These parameters are read by the code and a metadata output including error messages and the read-in parameters is generated within the terminal. All data outputs are in csv or txt format. A detailed description of each package and parameters is provided below.

#### region_size_and_abundance

*region_size_and_abundance* outputs the number of reads for each transcript feature (*i.e.* UTRs, ORF, start and stop codon) for different read lengths. We used the following settings for data analysis in this manuscript.

python region_size_and_abundance.py ../MANEv1.4_longnames.fasta

../80S_subset.SAM 80S_subset 1 25 34 4 none

python region_size_and_abundance.py ../MANEv1.4_longnames.fasta

../80S_subset.SAM 80S_subset 1 25 34 4 ./subset_list.xlsx

**Table:** 

Setting position	Setting name	Possible values	Explanation

0	*region_size_and_abundance.py*	N.A.	Python script that is used.

1	*fasta_in*	/path/to/ribofootprinter/MANEv1.4_longnames.fasta	Provide the path to the folder in which the MANE transcriptome is located

2	*sam_in*	/path/to/ribofootprinter/80S_subset.SAM	Provide the path to the folder in which the transcriptome aligned SAM files are located.

3	*outfile*	80S_subset	Provide a filename for your output file. An extension is not required.

4	*smallsize*	numerical	This setting sets the lower limit on read length. The minimal footprint length used in this study was 25 nt for the riboseq dataset.

5	*largesize*	numerical	This setting sets the upper limit on read length. The maximum footprint length used in this study was 34 nt for the riboseq dataset.

6	*window*	numerical	This setting determines the window around start and stop codon which defines the regions (start, stop, UTRs and CDS). A window of 4 was used in this study.

7	*subset_list*	none	No list is provided. All transcripts are used for analysis.
		/path/to/ribofootprinter/subset_list.xlsx	This file contains a list of transcripts for analysis. The first column contains the title *genenames* and corresponding ENSG gene names.

#### builddense

*builddense* converts SAM files into ROCC files which are required for multiple packages, including *writegene2, metagene, genelist, smorflist, posavg and posstats*. We used the following settings for data analysis in this manuscript, generating RPM normalized 5’-mapped ROCC files:

python builddense.py ./MANEv1.4_longnames.fasta ./80S_subset.SAM

80S_subset −1 25 34 1 > 80S_builddense_metadata.txt

python builddense.py./MANEv1.4_longnames.fasta ./40S_subset.SAM

40S_subset −1 20 80 1 > 40S_builddense_metadata.txt

**Table:** 

Setting position	Setting name	Possible values	Explanation

0	*builddense.py*	N.A.	Python script that is used.

1	*fasta_in*	/path/to/ribofootprinter/MANEv1.4_longnames.fasta	Provide the path to the folder in which the MANE transcriptome is located.

2	*sam_in*	/path/to/ribofootprinter/80S_subset.SAM	Provide the path to the folder in which the transcriptome aligned SAM files are located.

3	*outfile*	80S_subset	Provide a filename for your ROCC output file. An extension is not required.

4	*normalize*	−1	RPM normalization. The samples are normalized for sequencing depth. This allows comparison between samples.
		1E6	No normalization. Raw counts are used.
		numerical	Manually put in total reads to divide by for normalization.

5	*smallsize*	numerical	This setting sets the lower limit on read length

6	*largesize*	numerical	This setting sets the upper limit on read length.

7	*endmode*	1	Set to 1 for creating 5’-end mapped reads. 5’ endmode was used in this study.
		−1	Set to −1 for creating 3’-end mapped reads.
		0	Set to 0 for coverage (*e.g.* mRNA-seq data)

#### writegene2

writegene2 outputs ribosome profiling data aligned to a particular gene model. We used the following settings for data analysis in this manuscript, generating gene models for ACTB and EIF4G2 from ROCC files:

python writegene2.py ../80S_subset.rocc ACTB 80S_writegene2_ACTB

> 80S_writegene2_ACTB_metadata.txt

python writegene2.py ../80S_subset.rocc EIF4G2

80S_writegene2_EIF4G2 > 80S_writegene2_EIF4G2_metadata.txt

**Table:** 

Setting position	Setting name	Possible values	Explanation

0	*writegene2.py*	N.A.	Python script that is used.

1	*inputfiles*	/path/to/ribofootprinter/80S_subset.rocc	Provide the path to the folder in which the ROCC files are located. Multiple ROCC files can be provided simultaneously as a comma-separated list.

2	*genenames*	ACTB	Provide the genename. Multiple genenames can be provided simultaneously as a comma-separated list (*e.g*. “ACTB,GAPDH”) and will be written into a single output file.

3	*outfile*	80S_writegene2_ACTB	Provide a filename for your output file. An extension is not required.

#### genelist

*genelist* counts reads that map to CDS and UTR regions of gene models, and individual frames of the CDS. We used the following settings for data analysis in this manuscript:

python genelist.py ../80S_subset.rocc 12 1 80S_genelist >

80S_genelist_metadata.txt

python genelist.py ../40S_subset.rocc 12 1 40S_genelist >

40S_genelist_metadata.txt

**Table:** 

Setting position	Setting name	Possible values	Explanation

0	*genelist.py*	N.A.	Python script that is used.

1	*inputfiles*	/path/to/ribofootprinter/80S_subset.rocc	Provide the path to the folder in which the ROCC files are located

2	*shift*	numerical	This number indicates the shift used to align 5’-end mapped reads with the desired site within the ribosome. A shift of 12 typically matches the P-site of the ribosome.

3	*doextra*	0	No addition output file is generated.
		1	An additional output file containing ORF frame information for individual transcripts is generated.

4	*outfile*	80S_genelist	Provide a filename for your output file. An extension is not required.

#### metagene

metagene averages profiling data around start or stop codons. We used the following settings to generate a start and stop codon metagene in this study.

python metagene.py ../80S_subset.rocc 1 1 5 50 300 none

./80S_metagene_start_equalweight >

80S_metagene_start_metadata.txt

python metagene.py ../80S_subset.rocc 2 1 5 300 50 none

./80S_metagene_stop_equalweight > 80S_metagene_stop_metadata.txt

Setting to generate uORF-containing metagenes include:

python metagene.py ../80S_subset.rocc 1 1 5 50 300

subset_list.xlsx ./80S_metagene_start_equalweight_subset >

80S_metagene_start_metadata_subset.txt

python metagene.py ../80S_subset.rocc 2 1 5 300 50

subset_list.xlsx ./80S_metagene_stop_equalweight_subset >

80S_metagene_stop_metadata_subset.txt

**Table:** 

Setting position	Setting name	Possible values	Explanation

0	*metagene.py*	N.A.	Python script that is used.
1	*inputfiles*	/path/to/ribofootprinter/80S_subset.rocc	Provide the path to the folder in which the ROCC files are located.

2	*kind*	1	Metagene average around the start codon.
		2	Metagene average around the stop codon.

3	*weighting*	0	This setting makes an unweighted average based on RPM values.
		1	This setting equally weighs all genes.

4	*genetresh*	numerical	The minimal counts (RPKM) in the entire gene to be included in the analysis.

5	*range_5*	numerical	This setting determines the 5’-end range taken into account around the region of interest (start or stop codon).

6	*range_3*	numerical	This setting determines the 3”-end range taken into account around the region of interest (start or stop codon).

7	*subset_list*	none	No list is provided. All transcripts are used for analysis.
		/path/to/ribofootprinter/subset_list.xlsx	This file contains a list of transcripts for analysis. The first column contains the title *genenames* and corresponding ENSG gene names.

8	*outfile*	80S_metagene	Provide a filename for your output file. An extension is not required.

#### smorflist

*smorflist* counts reads that map in frame to uORF or dORFs in the 5’ or 3’ UTR, respectively. We used the following settings for data analysis in this manuscript identifying uORFs within the 5’-UTR of ribosome profiling data:

python smorflist.py ../80S_subset.rocc 4 12 0 0 5 80S_uorflist >

80S_uorflist_metadata.txt

**Table:** 

Setting position	Setting name	Possible values	Explanation

0	*smorflist.py*	N.A.	Python script that is used.

1	*inputfiles*	/path/to/ribofootprinter/80S_subset.rocc	Provide the path to the folder in which the ROCC files are located.

2	*lengththresh*	numerical	This is the threshold of number of amino acids within the uORF or dORF. For example 4 would report any uORF or dORF longer than 4 amino acids, while −4 would strickly report uORF or dORFs with a length of 4 amino acids.

3	*shift*	numerical	This number indicates the shift used to align 5’-end mapped reads with the desired site within the ribosome. A shift of 12 typically matches the P-site of the ribosome.

4	*smallest*	1	Keeps the smallest ORF (last start codon)
		0	Keeps the longest ORF

5	*mismatches*	1	This is how many mismatches to allow in start codon to allow identification of near start codon uORFs and dORFs (*i.e.* 1 mismatch allowed within the start codon sequence AUG).
		0	This setting allows no mismatches allowed within the start codon sequence AUG *(*i.e. only identifying uORFs and dORF starting with AUG).

6	*UTR*	5	This will run the script on the 5’-UTR identifying uORFs.
		3	This will run the script on the 3’-UTR identifying dORFs.

7	*outfile*	80S_smorflist	Provide a filename for your output file. An extension is not required.

#### posavg

posavg averages ribosome profiling data around any sequence feature of interest or computes a pause score for every occurrence of all 61 codons or 20 amino acids.

Pause score analysis comparing Pro and Ser and average pause score analysis:

python posavg.py ../80S_subset.rocc all 1 0 0 30 0 12 1 none

80S_posavg_all_aa_CDS_frame0 > 80S_posavg_all_aa _metadata.txt

uORF analysis with different normalization settings:

python posavg.py ../80S_subset.rocc ATG 0 3 0 30 0 13 0 none

80S_posavg_ATG_UTR5_allframe_norm0 >

80S_posavg_uORF_norm0_metadata.txt

python posavg.py ../80S_subset.rocc ATG 0 3 0 30 1 13 0 none

80S_posavg_ATG_UTR5_allframe_norm1 >

80S_posavg_uORF_norm1_metadata.txt

python posavg.py ../80S_subset.rocc ATG 0 3 0 30 0 13 2 none

80S_posavg_ATG_UTR3_allframe_norm0 >

80S_posavg_dORF_norm0_metadata.txt

python posavg.py ../80S_subset.rocc ATG 0 3 0 30 1 13 2 none

80S_posavg_ATG_UTR3_allframe_norm1 >

80S_posavg_dORF_norm1_metadata.txt

**Table:** 

Setting position	Setting name	Possible values	Explanation

0	*posavg.py*	N.A.	Python script that is used.

1	*inputfiles*	/path/to/ribofootprinter/80S_subset.rocc	Provide the path to the folder in which the ROCC files are located.

2	*motif (amino acid)*	P	Determines average around a single amino acid. Multiple amino acids can be provided simultaneously as a comma-separated list (e.g. “P,R,A”) and will be written into a single output file.
		PPG	Same as above but for amino acid motifs.
		all	All amino acids are calculated. Note this will also generate peak (pause) scores, using a hardcoded window of +/−1 nt. For the pause score all reads at the position of the peak +/−1 nt (nucleotide window of 3) are added together.
2	*motif (nucleotide)*	ATG	Determines average around a single nucleotide codon. Multiple codons can be provided simultaneously as a comma-separated list (e.g. “ATG,AAG,ACG”) and will be written into a single output file.
		all	All nucleotide triplets are calculated. Note this will also generate peak (pause) scores, using a hardcoded window of +/−1 nt.
		l or f	Put f or l at start of a motif to get the first or last such motif in the region of interest (note that all frames are checked).

3	*kind*	0	This indicates a nucleotide sequence.
		1	This indicates an amino acid

4	*frame*	0	Data will be analyzed for frame 0.
		1	Data will be analyzed for frame 1.
		2	Data will be analyzed for frame 2 (−1 frame)
		3	Data will be analyzed for all frames together.

5	*bkndwindowthresh*	numerical	The minimal RPKM counts within the bkndwindow to be included in the average.

6	*bkndwindow*	numerical	The size of the half-window around the codon of interest to be included in the average.

7	*ORFnorm*	0	No normalization.
		numerical	A positive value will ORF normalize (when calculating UTRs, UTRmode 0 or 2) and specify the rpkm ORF threshold for genes to include. This uses a hard-coded shift of +/−13.

8	*shift*	numerical	This number indicates the shift used to align 5’-end mapped reads with the desired site within the ribosome. A shift of 12 typically matches the P-site of the ribosome.

9	*UTRmode*	0	Data is analyzed for the 5’-UTR.
		1	Data is analyzed for the ORF.
		2	Data is analyzed for the 3’-UTR.

10	*subset_list*	none	No list is provided. All transcripts are used for analysis.
		/path/to/ribofootprinter/genelist.xlsx	This file contains a list of transcripts for analysis. The first column contains the title *genenames* and corresponding ENSG gene names.

11	*outfile*	80S_posavg	Provide a filename for your output file. An extension is not required.

#### posstats

*posstats* computes pause scores for every individual occurrence of a sequence feature of interest. We used the following settings for data analysis in this manuscript calculating pause scores for PPG and AAA in our ribosome profiling dataset. We used the R package ggplot2 to generate boxplots.

python posstats.py ../80S_subset.rocc PPG 1 0 10 1 12 1

p80S_posstats_PPG > 80S_posstats_PPG_metadata.txt

python posstats.py ../80S_subset.rocc AAA 1 0 10 1 12 1

80S_posstats_AAA > 80S_posstats_AAA_metadata.txt

**Table:** 

Setting position	Setting name	Possible values	Explanation

0	*posstats.py*	N.A.	Python script that is used.

1	*inputfiles*	/path/to/ribofootprinter/80S_subset.rocc	Provide the path to the folder in which the ROCC files are located.

2	*motif (amino acid)*	P	Determines average around a single amino acid.
		PPG	Same as above but for amino acid motif.
2	*motif (nucleotide)*	ATG	Determines average around a single nucleotide codon.

3	*kind*	0	This indicates a nucleotide seqeunce.
		1	This indicates that an amino acid motif.

4	*frame*	0	Data will be analyzed for frame 0.
		1	Data will be analyzed for frame 1.
		2	Data will be analyzed for frame 2 (−1 frame)

5	*genetresh*	numerical	The minimal RPKM counts in the entire gene to be included in the analysis.

6	*pkwindow*	numerical	How much on either side of the peak to use in numerator of pause score. For example, a pkwindow of 1 would count +/− 1 nucleotide (3 nucleotides) around the peak position. 0 or 1
			would be good starting values.

7	*shift*	numerical	Shift is a positive value for 5’ end aligned, negative for 3’ end aligned. It is amount to shift data before computing score (for example P site). A shift of 12 typically matches the P-site of the ribosome.

8	*UTRmode*	0	Data is analyzed for the 5’-UTR.
		1	Data is analyzed for the ORF.
		2	Data is analyzed for the 3’-UTR.

9	*outfile*	80S_posstats	Provide a filename for your output file. An extension is not required.

#### 3D_metagene

*3D_metagene* averages profiling data around start or stop codons for different read lengths. The read abundance is shown by a color scheme. The example shown below is to generate a start codon 3D metagene.

python 3D_metagene_5end.py ../MANEv1.4_longnames.fasta

../80S_subset.SAM ./80S_subset_3D.txt none 25 34 200 200 1 0

**Table:** 

Setting position	Setting name	Possible values	Explanation

0	*3D_metagene.py*	N.A.	Python script that is used.

1	*fasta_in*	/path/to/ribofootprinter/MANEv1.4_longnames.fasta	Provide the path to the folder in which the MANE transcriptome is located.

2	*sam_in*	/path/to/ribofootprinter/80S_subset. SA M	Provide the path to the folder in which the transcriptome aligned SAM files are located.

3	*outfile_path*	/path/to/ribofootprinter/80S_3D	Provide a filepath and name for your output file. An extension is not required.
4	*subset_list*	none	No list is provided. All transcripts are used for analysis.

		/path/to/ribofootprinter/subset_list. xlsx	This file contains a list of transcripts for analysis. The first column contains the title *genenames* and corresponding ENSG gene names.

5	*smallsize*	numerical	This setting sets the lower limit on read length

6	*largesize*	numerical	This setting sets the upper limit on read length.

7	*window_left*	numerical	This setting determines the window upstream of start or stop codon which defines the regions in which the metagene will be calculated

8	*window_right*	numerical	This setting determines the window downstream of start or stop codon which defines the regions in which the metagene will be calculated.

9	*metagene*	1	Generates 3D metagene around the start codon.
		2	Generates 3D metagene around the stop codon.

Plots can be generated from the *3D_metagene* output using the script below.

python 3D_metagene_plot.py ./80S_subset_3D.txt

### Datasets

We used two different types of datasets to provide examples for ribofootPrinter’s packages: 80S and 40S ribosome profiling. Libraries for both datasets were generated from A549 cells following a previously described protocol (McGlincy and Ingolia 2017). Prior to library preparation of the 40S samples, following steps were completed as described previously (Bohlen et al. 2020; Wagner et al. 2022) The cells were crosslinked prior to harvest. Following lysis, the ribosomes were separated on a 15%−35% sucrose gradient and fractions corresponding to the 40S subunit were collected. The RNA was reverse crosslinked and extracted by acid-phenol-chloroform extraction. Once RNA was obtained, the libraries were prepared similarly according to the standard protocol for Ribo-Seq. Size selection for 40S dataset was done for a 20–80 nt range while the 80S dataset was done for a 25–34 nt range.

Once libraries were prepared, they were sequenced as single-end reads on a Novaseq X 1.5B 100 cycle. The raw FASTQ files as described in (McGlincy and Ingolia 2017) to complete trimming, deduplication, and removal of contaminating non-coding reads. The footprint containing FASTQ files were then aligned against the reduced MANE v1.4 transcriptome (shortnames.FASTA) using following settings (*-v 1 -y -S*) resulting in a SAM file. To reduce the size of the SAM files, each dataset was reduced by randomly selecting only 50% of the reads.

samtools view -h 40S.SAM | awk ‘BEGIN {srand()} /^@/ {print}

!/^@/ {if (rand() <= 0.5) print}’ > 40S_subset.SAM

samtools view -h 80S.SAM | awk ‘BEGIN {srand()} /^@/ {print}

!/^@/ {if (rand() <= 0.5) print}’ > 80S_subset.SAM

### Visualizing datasets in IGV

As noted in the main text, a genome browser can be utilized to view the transcriptome aligned reads. We provide a guide on how to convert SAM files into IGV compatible files on the Github page. This includes conversion of SAM files into IGV-compatible BAM or BEDGRAPH/BIGWIG files. It also describes generation of a GTF file, which outlines the ORF boundaries for MANE v1.4 transcriptome transcripts, and is available for download.

Conversion of SAM to BAM files:

samtools sort -o 80S.bam 80S.SAM

samtools sort -o 40S.bam 40S.SAM

Indexing BAM files:

find *.bam -exec echo samtools index {} \; | sh

Conversion of BAM files to 5’-end aligned BEDGRAPH files:

for file in *.bam
do
     echo $file
     count=$(samtools view -F 4 $file | wc −l | xargs)
     cho $count
     scalefactor=$(echo “1000000 / $count” | bc −l)
     echo $scalefactor
     bedtools genomecov -ibam $file -scale $scalefactor -bg −5 >
${file%.bam}.bedgraph
done

Conversion of BAM files to uniform coverage BIGWIG files:

for file in *.bam
do
         echo $file
         bamCoverage -b ./$file -o ./${file%.bam}.bw -bs 1 --
normalizeUsing BPM
done

### Multimapping

#### Generation of transcriptome-derived FASTQ files

Transcriptome-derived FASTQ files containing every possible read that could be derived from the transcriptome sequences were generated using a custom Python script (*readgenerator_fullcov.py*). This script used the MANEv1.4_longnames reduced transcriptome file as input file and outputs FASTQ files with different read lengths as the setting *readlength* is adjusted. We generated a wide range of files containing footprints ranging from 10–34, 50, 75 and 100 nucleotides. Quality control on the generated FASTA was performed with the fastqc tool to determine correct read length. Full coverage of the FASTQ files was determined following bowtie alignment against the MANEv1.4_shortnames transcriptome and visualization of the BAM files in IGV. The number of unique reads within the full coverage transcriptome-derived FASTQ files was determined using the seqkit package (Shen et al. 2016):

seqkit rmdup -s | wc −l

Next, the full coverage transcriptome-derived FASTQ files were aligned against the shortnames transcriptome using different settings to determine the effect of footprint size (*i.e.* read length) on multimapping events.

mkdir -p nomatch

REF=path/to/bowtie_indexed/MANE_transcriptome/MANE_v1.4

for file in *.fq; do echo $file; bowtie -v 0 -y -S -p 12 -k 2 --

best --un ./nomatch/${file%.fq}_nomatch.fq -x $REF ./$file

./${file%.fq}.sam; done

The number of mismatches can be changed by adjusting *-v*. The -S setting outputs a SAM file. It is important to include the -k 2 setting which allows bowtie to report up to 2 alignments which is needed to identify multimapping events. Following alignment, bowtie will output information about read alignment. Subtracting the reads with at least one alignment from reported alignments gives the number of multimapped reads (this is possible because we only allow mapping to one other site by using the -k 2 setting). The percentage multimapped reads (y-axis in Figure 2D) can be calculated by dividing the number of multimapped reads by reads processed.

#### Generation of multimapper identifier files (mm_id)

The full coverage transcriptome-derived FASTQ files were used to identify multimapping regions. First, the FASTQ files were aligned against the reduced transcriptome using bowtie:

mkdir -p nomatch

REF=path/to/bowtie_indexed/MANE_transcriptome/MANE_v1.4

for file in *.fq; do echo $file; bowtie -v 0 -y -S -p 12 -m 1 --

best --norc --un ./nomatch/${file%.fq}_nomatch.fq -x $REF

./$file ./${file%.fq}.sam; done

The parameter *-m 1* instructs bowtie to only report unique alignments which allows us to divide the reads between uniquely mapped reads (exported as a SAM file, *- S* setting) and multimapped reads (exported as a nomatch FASTQ file, --*un ./nomatch/${file%.fq}_nomatch.fastq*). Once the nomatch FASTQ file containing multimapped reads has been obtained, they are realigned to the reduced transcriptome with bowtie settings allowing multiple multimapped reads *(-k 100000* setting). This will output a SAM file containing information on exclusively multimapped reads.

cd nomatch

REF=path/to/bowtie_indexed/MANE_transcriptome/MANE_v1.4

for file in *.fq; do echo $file; bowtie -v 0 -y -S -p 12 -k

100000 --best --norc -x $REF ./$file ./${file%.fq}.sam; done

The SAM file containing multimapped reads is converted into 5’-end aligned bedgraph files using the *samtools* and *bedtools* package.

for file in *.sam; do samtools sort -o ${file%.sam}.bam $file;

done

for file in *.bam; do echo $file; bedtools genomecov -ibam $file

-bg −5 > ${file%.bam}_mm_id.bedgraph; done

These multimapper identified (mm_id.bedgraph) files can be viewed in IGV together with aligned reads of interest. The metric encoded in these files is therefore 0 for positions that map uniquely. Otherwise, the value indicates the number of sites the read could map to, and is capped at 100,000 sites. Since most cases of multiple mapping involve a few sites, it is advisable to set the axis limits to <10 when viewing these files.

### Clustal Omega protein alignment

Nucleotide sequences in fasta format for *ACTB, POTEI* and *POTEJ* were obtained from the MANEv1.4_longnames.FASTA file. These sequences were aligned using the Multiple Sequence Alignment (MSA) tool from Clustal Omega (Madeira et al. 2024) using settings (Sequence type: *RNA,* Parameters: *ClustalW with character counts*).

## Supplementary Material

Supplement 1
