# Supplementary material for: ribofootPrinter: A precision python toolbox for analysis of ribosome profiling data
Source: bioRxiv. 2025 Sep 15:2021.07.04.451082. Preprint. [Version 2] doi: 10.1101/2021.07.04.451082 (PMC12458181; doi:10.1101/2021.07.04.451082)
Supplement: Supplement 1 [file NIHPP2021.07.04.451082v2-supplement-1.pdf]

bioRxiv preprint doi: https://doi.org/10.1101/2021.07.04.451082; this version posted September 15, 2025. The copyright holder for this preprint (which was not certified by peer review) is the author/funder. This article is a US Government work. It is not subject to copyright under 17 USC 105 and is also made available for use under a CC0 license.

Supplementary Figure Legends

Supplementary Figure 1. Overview of additional information provided to enhance ribofootPrinter usability.

(A) The MANE transcriptome for bowtie alignments (shortnames) or ribofootPrinter (longnames) can be created using commands that are provided.

(B) Description of the pipeline used to convert aligned SAM file into genome browser compatible files.

Supplementary Figure 2. Generation of multimapper identifier (mm_id) files.

The transcriptome-derived FASTQ files were aligned against the transcriptome using bowtie with settings only allowing uniquely mapped reads (*-m 1*). This results in a SAM file containing uniquely mapped reads only and a FASTQ file with unmapped reads (containing multimappers). The FASTQ file containing non-uniquely aligned reads is aligned against the reduced transcriptome again with bowtie using a setting allowing a large number of multimapping events (*-k 100000*). This generates a SAM file containing multimapped reads which can be converted into IGV compatible BEDGRAPH files termed multimapper identifier files.

Supplementary Figure 3. Algorithm used to determine footprint length distribution within UTRs, ORF, start or stop codon.

The *region_size_and_abundance* package defines a window around the start and stop codon which will determine the UTR and ORF boundaries. A read is counted as start or

bioRxiv preprint doi: https://doi.org/10.1101/2021.07.04.451082; this version posted September 15, 2025. The copyright holder for this preprint (which was not certified by peer review) is the author/funder. This article is a US Government work. It is not subject to copyright under 17 USC 105 and is also made available for use under a CC0 license.

stop if the read overlaps with the start or stop codon region. Only partially overlapping reads are distributed into UTR or ORF, depending on their location. This is shown as a cartoon (top). The two abundance calculations can be used to normalize the output probability density and then create two normalized histograms (bottom).

Supplementary Figure 4. Algorithm used to generate metagene plots.

(A) Metagene plots are generated by aligning the mapped reads around the start or stop codon, followed by taking the average across all transcripts. This reveals nucleotide periodicity within the ORF for riboseq data.

(B) Schematic representation of the ribosome protecting a fixed number of nucleotides (this can differ depending on the sample preparation method). Different types of analysis can be completed for 5’- or 3’-end aligned reads. If desired, shifts can be calculated to study the E-, P- or A- sites.
